# Real-world experience of nintedanib for progressive fibrosing interstitial lung disease in the UK

**DOI:** 10.1183/23120541.00529-2023

**Published:** 2024-01-15

**Authors:** Giles Dixon, Samuel Hague, Sarah Mulholland, Huzaifa Adamali, Aye Myat Noe Khin, Hannah Thould, Roisin Connon, Paul Minnis, Eoin Murtagh, Fasihul Khan, Sameen Toor, Alexandra Lawrence, Marium Naqvi, Alex West, Robina K. Coker, Katie Ward, Leda Yazbeck, Simon Hart, Theresa Garfoot, Kate Newman, Pilar Rivera-Ortega, Lachlan Stranks, Paul Beirne, Jessica Bradley, Catherine Rowan, Sarah Agnew, Mahin Ahmad, Lisa G. Spencer, Joshua Aigbirior, Ahmed Fahim, Andrew M. Wilson, Elizabeth Butcher, Sy Giin Chong, Gauri Saini, Sabrina Zulfikar, Felix Chua, Peter M. George, Maria Kokosi, Vasileios Kouranos, Philip Molyneaux, Elisabetta Renzoni, Benedetta Vitri, Athol U. Wells, Lisa M. Nicol, Stephen Bianchi, Raman Kular, HuaJian Liu, Alexander John, Sarah Barth, Melissa Wickremasinghe, Ian A. Forrest, Ian Grimes, A. John Simpson, Sophie V. Fletcher, Mark G. Jones, Emma Kinsella, Jennifer Naftel, Nicola Wood, Jodie Chalmers, Anjali Crawshaw, Louise E. Crowley, Davinder Dosanjh, Christopher C. Huntley, Gareth I. Walters, Timothy Gatheral, Catherine Plum, Shiva Bikmalla, Raja Muthusami, Helen Stone, Jonathan C.L. Rodrigues, Krasimira Tsaneva-Atanasova, Chris J. Scotton, Michael A. Gibbons, Shaney L. Barratt

**Affiliations:** 1 Bristol Interstitial Lung Disease Service, North Bristol NHS Trust, Bristol, UK; 2 South West Peninsula ILD Network, Royal Devon University Healthcare NHS Foundation Trust, Exeter, UK; 3 Department of Clinical and Biomedical Sciences, University of Exeter, Exeter, UK; 4 Royal United Hospitals Bath NHS Foundation Trust, Bath, UK; 5 Antrim Area Hospital, Northern Health and Social Care Trust, Antrim, UK; 6 Glenfield Hospital, University Hospitals of Leicester NHS Trust, Leicester, UK; 7 Guy's and St Thomas’ Hospital NHS Foundation Trust, London, UK; 8 Hammersmith Hospital, Imperial College Healthcare NHS Trust, London, UK; 9 Hull University Teaching Hospitals NHS Trust, Hull, UK; 10 Interstitial Lung Disease Unit, Wythenshawe Hospital, Manchester University NHS Foundation Trust, Manchester, UK; 11 Leeds Teaching Hospitals NHS Trust, Leeds, UK; 12 Liverpool Interstitial Lung Disease Service, Aintree Hospital, Liverpool University Hospital NHS FT, Liverpool, UK; 13 New Cross Hospital, The Royal Wolverhampton NHS Trust, Wolverhampton, UK; 14 Norfolk and Norwich University Hospital NHS Foundation Trust, UK; 15 Norwich Medical School, University of East Anglia, Norwich, UK; 16 Nottingham University Hospitals NHS Trust, Nottingham, UK; 17 Oxford University Hospitals NHS Foundation Trust, Oxford, UK; 18 Royal Brompton and Harefield Hospitals, London, UK; 19 Royal Infirmary of Edinburgh, Edinburgh, UK; 20 Sheffield Teaching Hospital NHS Foundation Trust, Sheffield, UK; 21 Southern Health and Social Care Trust, Portadown, UK; 22 St Mary's Hospital, Imperial College Healthcare NHS Trust, London, UK; 23 The Newcastle upon Tyne Hospitals NHS Foundation Trust, Newcastle, UK; 24 Newcastle University, Newcastle upon Tyne, UK; 25 University Hospital of Southampton NHS Foundation Trust, Southampton, UK; 26 NIHR Southampton Respiratory Biomedical Research Centre and School of Clinical and Experimental Sciences, Faulty of Medicine, University of Southampton, Southampton, UK; 27 University Hospitals Birmingham NHS Foundation Trust, Birmingham, UK; 28 Institute of Applied Health Research, University of Birmingham, Birmingham, UK; 29 University Hospitals of Morecambe Bay NHS Foundation Trust, Lancashire and South Cumbria ILD Service, Lancaster, UK; 30 University Hospitals of North Midlands NHS Trust, Stoke-on-Trent, UK; 31 Department of Health, University of Bath, Bath, UK; 32 Department of Mathematics and Statistics, Faculty of Environment, Science and Economy, University of Exeter, Exeter, UK; 33 EPSRC Hub for Quantitative Modelling in Healthcare, University of Exeter, Exeter, UK; 34 Living Systems Institute, University of Exeter, Exeter, UK; 35 These authors contributed equally

## Abstract

**Background:**

Nintedanib slows progression of lung function decline in patients with progressive fibrosing (PF) interstitial lung disease (ILD) and was recommended for this indication within the United Kingdom (UK) National Health Service in Scotland in June 2021 and in England, Wales and Northern Ireland in November 2021. To date, there has been no national evaluation of the use of nintedanib for PF-ILD in a real-world setting.

**Methods:**

26 UK centres were invited to take part in a national service evaluation between 17 November 2021 and 30 September 2022. Summary data regarding underlying diagnosis, pulmonary function tests, diagnostic criteria, radiological appearance, concurrent immunosuppressive therapy and drug tolerability were collected *via* electronic survey.

**Results:**

24 UK prescribing centres responded to the service evaluation invitation. Between 17 November 2021 and 30 September 2022, 1120 patients received a multidisciplinary team recommendation to commence nintedanib for PF-ILD. The most common underlying diagnoses were hypersensitivity pneumonitis (298 out of 1120, 26.6%), connective tissue disease associated ILD (197 out of 1120, 17.6%), rheumatoid arthritis associated ILD (180 out of 1120, 16.0%), idiopathic nonspecific interstitial pneumonia (125 out of 1120, 11.1%) and unclassifiable ILD (100 out of 1120, 8.9%). Of these, 54.4% (609 out of 1120) were receiving concomitant corticosteroids, 355 (31.7%) out of 1120 were receiving concomitant mycophenolate mofetil and 340 (30.3%) out of 1120 were receiving another immunosuppressive/modulatory therapy. Radiological progression of ILD combined with worsening respiratory symptoms was the most common reason for the diagnosis of PF-ILD.

**Conclusion:**

We have demonstrated the use of nintedanib for the treatment of PF-ILD across a broad range of underlying conditions. Nintedanib is frequently co-prescribed alongside immunosuppressive and immunomodulatory therapy. The use of nintedanib for the treatment of PF-ILD has demonstrated acceptable tolerability in a real-world setting.

## Tweetable abstract

**Nintedanib is used for the treatment of PF-ILD in the UK for a broad range of underlying diagnoses and with variation in disease severity at the point of initiation. Real-world data suggest an acceptable tolerability profile of nintedanib in PF-ILD.** https://bit.ly/3Mdmtri

## Introduction

Progressive fibrosing (PF) interstitial lung disease (ILD) describes a cohort of patients who develop disease progression despite optimal pharmacotherapy [1]. It is characterised by a combination of worsening respiratory symptoms, declining lung function and increasing extent of fibrosis on high-resolution computed tomography (HRCT). PF-ILD is observed in a wide range of fibrosing ILDs including connective tissue disease (CTD)-associated ILD, hypersensitivity pneumonitis, idiopathic nonspecific interstitial pneumonia (NSIP) and unclassifiable ILD [1]. The clinicophenotypical and mechanistic overlap between idiopathic pulmonary fibrosis (IPF) and PF-ILD allows the potential for a common treatment pathway [1].

Estimations of the proportion of patients with fibrosing ILD who develop a progressive phenotype have varied historically, and have been reported to be between 13% and 53% [2]. Recently, a robust multicentre Canadian prospective registry study has demonstrated progression in 39–59% of patients with fibrosing ILD despite conventional therapy, dependent on disease subtype [3].

The landmark INBUILD study demonstrated the efficacy of the antifibrotic tyrosine kinase inhibitor nintedanib in treating a wide range of fibrosing ILDs [4]. Nintedanib was shown to reduce the annual rate of lung function decline in patients with fibrosing ILD regardless of underlying subtype [4–6]. Nintedanib was approved by the National Institute of Health and Care Excellence (NICE) in November 2021 for use in PF-ILD. The NICE technology appraisal used the criteria established by the INBUILD study to define the prescribing criteria in England, Wales and Northern Ireland [4].

Nintedanib is licensed for use in patients with fibrosing ILD which has progressed despite conventional therapy. While the INBUILD study excluded the use of immunosuppression other than low-dose prednisolone at baseline, evidence for the concomitant use of nintedanib and immunosuppression originates from the Safety and Efficacy of Nintedanib in Systemic Sclerosis (SENSCIS) study of nintedanib in systemic sclerosis-associated ILD. The study included 279 (48.4%) out of 576 participants who were receiving mycophenolate mofetil (MMF) at baseline [6]. Nintedanib reduced the annual rate of forced vital capacity (FVC) decline in participants both receiving and not receiving MMF, with no difference in adverse events [7].

Data from a national early access programme in the United Kingdom (UK) demonstrated real-world efficacy of nintedanib in PF-ILD [8]. Despite the study cohort demonstrating a greater impairment in FVC and transfer capacity of the lung for carbon monoxide (*D*_LCO_) at baseline compared to INBUILD, nintedanib was still able to slow the rate of lung function decline.

The aim of this UK-wide service evaluation was to document prescribing practices in the real world for the use of nintedanib for PF-ILD. We aimed to document the criteria used for PF-ILD diagnosis, the underlying disease subsets, the severity of disease at drug initiation and the concomitant use of immunosuppressive therapies.

## Methods

### Service evaluation

In England, antifibrotic medications are available through ILD specialist centres and through general hospitals in Scotland, Wales and Northern Ireland. 26 antifibrotic prescribing centres in the UK were invited to service evaluation inclusion by email. Individual participating centres registered this project with their local healthcare trust service evaluation/audit departments, complying with Caldicott principles. No personal identifiable information was submitted for study. The study was not considered research by the UK Health Research Authority (HRA) decision tool and did not require HRA or ethical approval.

### Data collection

Study centres completed a pre-defined survey for patients with a multidisciplinary team (MDT) decision to commence nintedanib for non-IPF PF-ILD between 17 November 2021 and 30 September 2022. A copy of the survey in presented in the supplementary material.

Data collected included underlying diagnosis, diagnostic criteria, concomitant therapy, radiological pattern, FVC and *D*_LCO_ at baseline and reason for drug discontinuation.

Summary data were grouped into categories prior to submission by individual participating centres. Individual-level patient data were not collated centrally.

Responses were collected by electronic survey (Jisc Online Surveys, UK) and collated by the coordinating centres (North Bristol NHS Trust and Royal Devon University Healthcare NHS Foundation Trust).

### Progression criteria

The UK has adopted progression criteria defined by the INBUILD study [4]. All patients commenced upon nintedanib for PF-ILD are required to fulfil one of the following criteria:

1. a relative decline in FVC of ≥10% predicted over the previous 24 months;
2. a relative decline in FVC% predicted of ≥5%, but <10%, with worsening respiratory symptoms;
3. a relative decline in FVC% predicted of ≥5%, but <10%, with increasing fibrotic changes on HRCT compared with the previous 24 months;
4. worsening respiratory symptoms and increasing fibrotic changes on HRCT over the previous 24 months.

## Results

24 (92.3%) out of 26 centres responded to the survey; participating centres are listed in the supplementary material. The number of patients prescribed nintedanib across specialist centres differed during the assessment period (between seven and 129). In total, 1120 patients had an MDT recommendation to commence nintedanib for PF-ILD.

### Treatment by subtype

**FIGURE 1:**
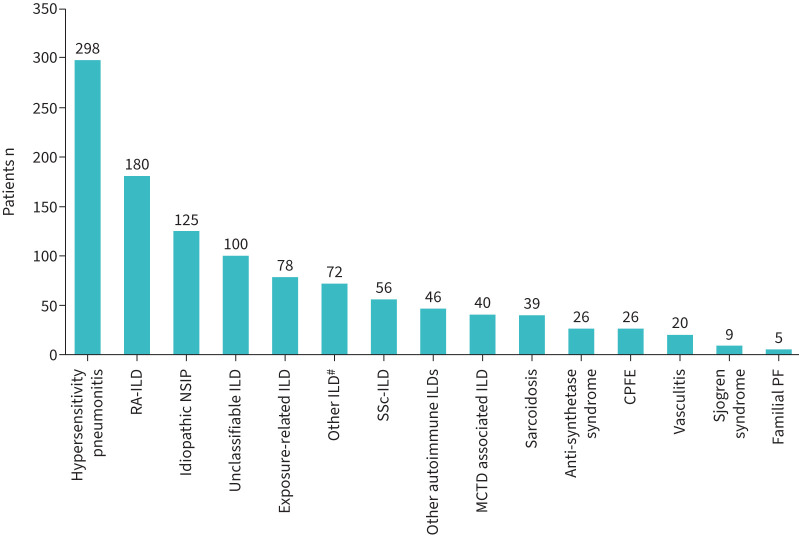
Primary diagnosis of 1120 patients with an interstitial lung disease (ILD) multidisciplinary team decision to commence nintedanib between 17 November 2021 and 30 September 2022. RA: rheumatoid arthritis; NSIP: nonspecific interstitial pneumonia; SSc: systemic sclerosis; MCTD: mixed connective tissue disease; CPFE: combined pulmonary fibrosis and emphysema syndrome; PF: pulmonary fibrosis. ^#^: the predominant diagnoses included in “other ILD” were pleuroparenchymal fibroelastosis in 19 (1.7%) out of 1120, other connective tissue disease-related ILD in 11 (1.0%) out of 1120, fibrotic organising pneumonia in seven (0.6%) out of 1120, interstitial pneumonia with autoimmune features in six (0.5%) out of 1120, asbestosis in five (0.4%) out of 1120, desquamative interstitial pneumonia in four (0.4%) out of 1120, post-coronavirus disease 2019 ILD in three (0.3%) out of 1120 and sarcoidosis in two (0.2%) out of 1120.

Figure 1 demonstrates the subtypes of ILD for which nintedanib was prescribed. The most common subtypes were hypersensitivity pneumonitis (298 out of 1120, 26.6%), rheumatoid arthritis related ILD (180 out of 1120, 16.0%), idiopathic NSIP (125 out of 1120, 11.2%) and unclassifiable ILD (100 out of 1120, 8.9%). 72 out of 1120 patients had criteria for diagnosis labelled as “other”. This included pleuroparenchymal fibroelastosis (PPFE) (19 out of 1120, 1.7%), other CTD-related ILD (11 out of 1120, 1.0%), fibrotic organising pneumonia (seven out of 1120, 0.6%), interstitial pneumonia with autoimmune features (six out of 1120, 0.5%) and asbestosis (five out of 1120, 0.4%).

### PF-ILD criteria and radiological pattern

**FIGURE 2:**
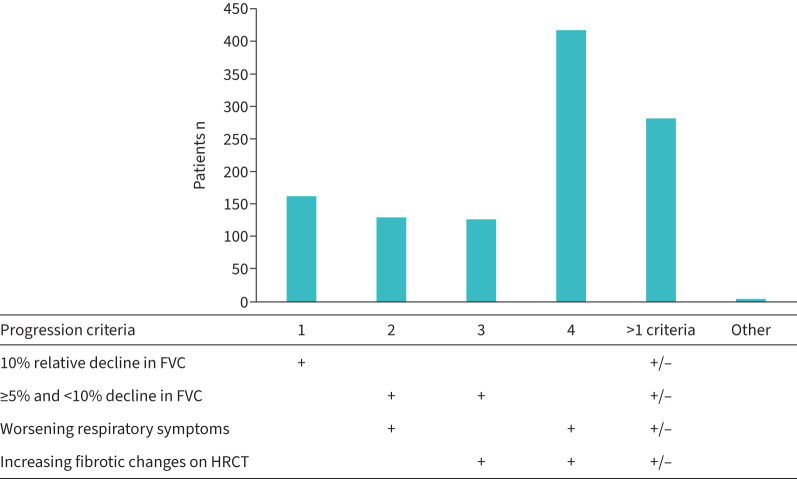
Diagnostic criteria for progressive fibrosing interstitial lung disease (ILD) in 1120 patients with an ILD multidisciplinary team decision to commence nintedanib between 17 November 2021 and 30 September 2022. FVC: forced vital capacity; HRCT: high-resolution computed tomography.

Figure 2 demonstrates the primary criteria by which PF-ILD was diagnosed in the cohort. 418 (37.3%) out of 1120 were diagnosed based on progressive disease identified on HRCT with progression of symptoms (criterion 4). 281 (25.1%) out of 1120 patients fulfilled more than one diagnostic criterion for nintedanib prescription.

The MDT consensus radiological patterns reported were definite usual interstitial pneumonia (UIP) pattern (252 out of 1120, 22.5%), probable UIP (111 out of 1120, 9.9%), indeterminate for UIP (53 out of 1120, 4.7%), fibrotic hypersensitivity pneumonitis (262 out of 1120, 23.4%), fibrotic NSIP (261 out of 1120, 23.3%) and alternative pattern (181 out of 1120, 16.2%).

### Concomitant therapy

Concomitant immunomodulatory therapy was commonly prescribed. 609 (54.4%) out of 1120 patients were receiving oral corticosteroids at the time of commencing nintedanib. MMF was the most commonly co-prescribed immunosuppressive therapy after corticosteroids (335 out of 1120, 29.9%). Table 1 demonstrates the range of immunomodulatory and immunosuppressive therapies that were intended to be continued alongside nintedanib.

**TABLE 1:** Intended concomitant prescription of immunomodulatory or immunosuppressive therapy with nintedanib for progressive fibrosing interstitial lung disease

**Oral corticosteroids^#^**	609 (54.4)
**Mycophenolate mofetil**	355 (31.7)
**Hydroxychloroquine**	76 (6.8)
**Methotrexate**	73 (6.5)
**Rituximab**	70 (6.3)
**Azathioprine**	36 (3.2)
**Sulfasalazine**	36 (3.2)
**Cyclophosphamide**	13 (1.2)
**Abatacept**	6 (0.5)
**Tacrolimus**	6 (0.5)
**Leflunomide**	5 (0.4)
**Tocilizumab**	5 (0.4)
**Etanercept**	3 (0.3)
**Other^¶^**	11 (1.0)

Data are presented as n (%). **^#^**: ≥10 mg prednisolone or equivalent; ^¶^: included adalimumab, denosumab, baracitinib, intravenous immunoglobulin, sildenafil, upadacitinib and certolizumab.

Immunosuppressive or immunomodulatory therapy was stopped prior to commencing nintedanib in 21 out of 1120 patients. The most commonly discontinued medications were MMF (eight out of 21) and oral corticosteroids (five out of 21).

### Pulmonary function at time of initiation

**FIGURE 3:**
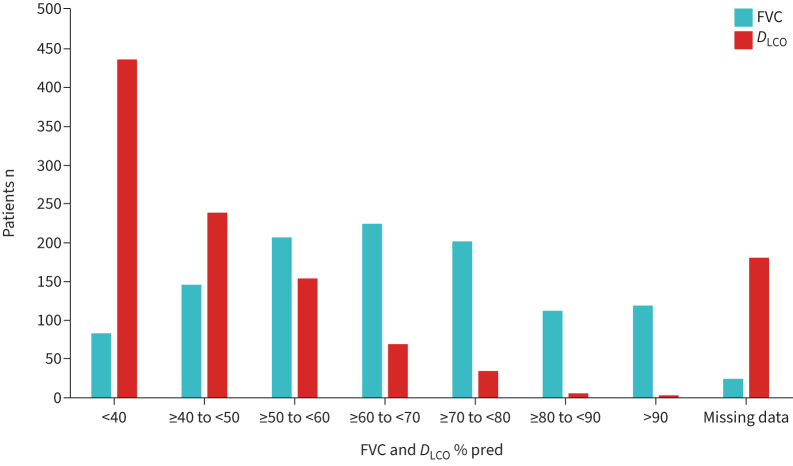
Percentage predicted forced vital capacity (FVC) and transfer capacity of the lung for carbon monoxide (*D*_LCO_) for 1120 patients with an interstitial lung disease multidisciplinary team decision decision to commence nintedanib between 17 November 2021 and 30 September 2022.

Baseline pulmonary function tests at the time of MDT decision to commence nintedanib for PF-ILD are demonstrated in figure 3. The median percentage predicted FVC category was ≥60% pred to <70% pred and median percentage predicted *D*_LCO_ category was <40%. 181 out of 1120 participants had no value for percentage predicted *D*_LCO_, which represented patients with missing data or who were unable to perform *D*_LCO_ testing.

### Multidisciplinary team

There were a range of prescribing healthcare professionals (n=24) across and within prescribing centres. The healthcare professionals prescribing nintedanib were reported to be respiratory physicians (23 out of 24), nurse specialists (14 out of 24), specialist pharmacists (12 out of 24) and rheumatologists (four out of 24).

### Drug initiation and discontinuation

By 30 September 2022, 928 (82.9%) out of 1120 patients had commenced nintedanib; the remaining patients were awaiting initiation. The proportion of patients awaiting drug initiation as of 30 September 2022 varied by prescribing centre, ranging from 42% to 100% of patients. 10 out of 24 participating centres had initiated all intended patients on nintedanib by 30 September 2022.

At the time of service evaluation submission, 175 (18.8%) out of 928 participants had discontinued nintedanib. The most common reasons for nintedanib discontinuation were death (63 out of 175, 36.0%), drug tolerability (83 out of 175, 47.4%) and deranged liver function test (16 out of 175, 9.1%).

## Discussion

The service evaluation has demonstrated widespread uptake of the use of nintedanib for PF-ILD in the UK. The NICE technology appraisal guidance predicted that a total of 900 patients in the UK living with PF-ILD would be eligible for nintedanib [9]. This service evaluation has demonstrated that 1120 patients had an MDT decision to commence nintedanib for PF-ILD between November 2021 and September 2022 and 928 had initiated treatment by 30 September 2022. This highlights the underestimation of the potentially eligible patients living with PF-ILD in the UK, which has important implications for service provision.

The study highlights variation in the proportion of patients with an MDT decision to commence nintedanib and those who have initiated treatment. This variation could be explained by a combination of size of specialist centre, local referral patterns and the ability of individual centres to manage the increased demand for nintedanib initiation. Discrepancies in UK service provision have been highlighted by the 2021 Getting it Right First Time report [10]. This report revealed variation in waiting times for clinical assessment, differences in medical workforce provision and variation in specialist nursing and pharmacy services. The prescription of nintedanib for PF-ILD in the UK was not permitted until 90 days following the NICE recommendation. This had the effect of reducing the period covered by this evaluation within which patients could commence treatment.

Our data identified fewer patients commenced on nintedanib for lung function deterioration compared to the INBUILD study [4]. A relative decline in FVC of >10% pred was the most common criterion met to diagnose PF-ILD in the INBUILD treatment arm (160 out of 332, 48.2%) compared to only 162 (14.5%) out of 1120 in our study. Our study demonstrated a higher proportion of patients having progression defined by HRCT (418 out of 1120, 37.3%) than those in the INBUILD treatment arm (n=62, 18.7%). FVC trajectories are known to be poorer in those with disease progression identified by HRCT and our data demonstrate the real-world practice of using HRCT to determine disease progression prior to treatment initiation [11]. This increased reliance on HRCT could reflect the reduced availability of lung function testing during the coronavirus disease 2019 pandemic [12]. Regardless of the rationale, the increased use of HRCT to diagnose progression emphasises the requirement for specialist thoracic radiologist review in the context of an MDT. Accurate quantification of disease progression including the identification of subtle changes in disease extent and morphology may be aided by the use of artificial intelligence assessment of serial HRCT [13].

PF-ILD encompasses a broad range of underlying ILD subtypes. This service evaluation highlights differences between our real-world patient cohort and that examined by the INBUILD study. A higher proportion of autoimmune ILDs (including rheumatoid arthritis-associated ILD and CTD-ILD) were seen in this service evaluation compared to the INBUILD nintedanib arm (377 (33.7%) out of 1120 and 82 (24.7%) out of 332, respectively), perhaps reflecting the exclusion of patients receiving concomitant immunosuppression in INBUILD [4]. Idiopathic NSIP and unclassifiable ILDs were underrepresented in the real-world study compared to INBUILD; 125 (11.1%) out of 1120 *versus* 64 (19.3%) out of 332 and 100 (8.9%) out of 1120 *versus* 64 (19.3%) out of 332, respectively. However, there were significant similarities, for example hypersensitivity pneumonitis was the most common underlying diagnosis in both the INBUILD nintedanib arm (84 out of 332, 25.3%) and our service evaluation (298 out of 1120, 26.7%).

These data have highlighted the use of nintedanib for PPFE (19 out of 1120, 1.7%). Both PPFE and nintedanib use are associated with weight loss and the nintedanib rate of adverse events of in this population is unknown [14, 15]. Evidence for the use of nintedanib in PPFE is limited to conflicting retrospective reports, highlighting the need for prospective, controlled studies [16, 17]. While the INBUILD study did include patients with PPFE, they represented a small proportion of the overall cohort and were not analysed as a separate subgroup [18]. Patients being commenced on nintedanib for PPFE will require close monitoring and follow-up to manage potentially burdensome adverse events.

Nintedanib has a broad range of antifibrotic, anti-inflammatory and vascular remodelling effects [19]. Immunosuppressive therapies have a myriad of mechanisms of action depending on the drug in question, different to those seen with nintedanib therapy. Combined immunosuppressive and antifibrotic treatment may therefore offer synergistic effects of reducing the progression of ILD. Despite this promise there are limited trial or real-world data concerning the efficacy of co-prescription of these drugs [8]. Our data have highlighted the common use of nintedanib combined with immunosuppressive or modulatory therapies (table 1). Oral corticosteroids (609 out of 1120, 54.4%), MMF (335 out of 1120, 29.9%), hydroxychloroquine (76 out of 1120, 6.8%) and methotrexate (73 out of 1120, 6.5%) were all commonly used alongside nintedanib.

The INBUILD trial did not include participants taking concomitant immunosuppressive therapy, except for low-dose corticosteroids; however, 16% of participants were initiated on therapy other than nintedanib after 6 months [4]. In the treatment arm of the SENSCIS trial of nintedanib for systemic sclerosis, 48% (139 out of 288) of patients were receiving MMF with a suggested beneficial effect of MMF on lung function decline and without increased adverse events when used in combination with nintedanib. However, the seminal PANTHER study of immunosuppression in IPF demonstrated the potential harmful effect of immunosuppression in patients with IPF. Consequently, the use of immunosuppression in the context of a progressive fibrosing phenotype with UIP pattern fibrosis requires further evidence to ensure greatest patient benefit and minimise potential harm [20].

Within the SENSCIS trial adverse event rate in the nintedanib and placebo arms were similar between the subgroups receiving and not receiving MMF [7]. The study reported that 15 (10.8%) out of 139 participants in the nintedanib group who were receiving MMF at baseline discontinued nintedanib treatment. By comparison, we demonstrated an overall discontinuation rate for reasons other than death in 112 (12.1%) out of 928 patients. Limited real-world single-centre tolerability data demonstrated no significant difference in nintedanib discontinuation rates, tolerability or side-effect profile between cohorts receiving co-antifibrotic and immunosuppressive therapy and those receiving antifibrotic monotherapy [21].

The overall discontinuation rate in our cohort (including death) was 175 (18.9%) out of 928. Real-world UK data for the use of nintedanib in IPF demonstrate varying overall discontinuation rates ranging from 26% (32 out of 119) to 30% (15 out of 49) [22, 23]. The discrepancies between these data and those reported for our cohort may represent several factors including the shorter follow-up period in our service evaluation, improvements in adverse-effect management and differences in patient demographics and disease severity. It is encouraging that our real-world data suggest an acceptable tolerability profile of nintedanib in PF-ILD.

Importantly, 63 (6.8%) out of 928 patients had death recorded as the reason for nintedanib discontinuation. The limitations of the data collected mean that we are unable to identify the duration of treatment with nintedanib prior to death. However, the study does identify a high proportion of patients commencing nintedanib with significant lung function impairment. 231 (20.6%) out of 1120 had an FVC <50% pred at the time of MDT decision to commence nintedanib, and 436 (38.9%) out of 1120 had a *D*_LCO_ <40% pred. This could reflect the recent approval of nintedanib for patients who previously had no antifibrotic treatment options and therefore had more advanced disease at the time of initiation. While the unpredictable nature of ILD progression makes prognostication difficult, consideration of life expectancy and severity of lung function impairment should be considered prior to initiation of nintedanib to ensure that the beneficial effect on lung function preservation remains greater than the symptom burden. In patients with high symptom burden and poor prognosis, a supportive management approach may be more appropriate.

### Limitations

There are several limitations to the reported service evaluation. Firstly, the service evaluation methodology was adopted to enable rapid data collection; as such, the study did not record individual-level patient data. This limited the interpretation of individual patient prescribing patterns. In addition, the doses of oral corticosteroids and immunosuppression were not recorded. The data did not capture patients who were, for example, prescribed oral corticosteroids and MMF with nintedanib. We are unable to elucidate whether those patients who discontinued nintedanib treatment were those on concomitant therapy or had greater impairment in lung function. In addition, the specific drug tolerability reasons were not recorded as part of the study.

The NICE guidance is only applicable to NHS England, Wales and Northern Ireland. Nintedanib was available for PF-ILD prior to November 2021 in NHS Scotland. Our service evaluation may not have captured cohorts of patients commenced on nintedanib prior to this date. Furthermore, patients were able to receive nintedanib on a named-patient basis prior to November 2021, as reported by Raman *et al*. [8]. These patients were purposely excluded from the current study, but represent an important cohort of patients with PF-ILD who may benefit from nintedanib. There will also be a cohort of patients who meet PF-ILD diagnostic criteria, but decline or have contraindications to antifibrotic treatment. This cohort was not captured by the current study.

Despite these limitations, this study presents real-world data for the majority of UK prescribing centres and provides important practice-based evidence for the management of PF-ILD.

### Conclusions

Nintedanib is widely prescribed in UK practice for the treatment of PF-ILD. Our service evaluation has demonstrated its use in a variety of underlying diagnoses, for a broad range of disease severity and commonly with concomitant immunosuppressive therapy. The service evaluation has highlighted variances in prescribing practices and important distinctions between real-world and clinical trial practice. Furthermore, we have emphasised gaps in the evidence base including the use of concomitant immunosuppression and antifibrotic therapy, the use of nintedanib in patients with severely impaired lung function and the increased use of HRCT to identify disease progression.

## Supplementary material

10.1183/23120541.00529-2023.Supp1 **Please note:** supplementary material is not edited by the Editorial Office, and is uploaded as it has been supplied by the author.
Questionnaire 00529-2023.SUPPLEMENT
Coordinating and participating 00529-2023.SUPPLEMENT2
