# Supplementary material for: Real-world experience of nintedanib for progressive fibrosing interstitial lung disease in the UK
Source: ERJ Open Res. 2024 Jan 15;10(1):00529-2023. doi: 10.1183/23120541.00529-2023 (PMC10789269; doi:10.1183/23120541.00529-2023)
Supplement: Supplementary file 1 [file 00529-2023.SUPPLEMENT.pdf]

UK PF-ILD Nintedanib Service Evaluation

Page 1: A service evaluation of the UK Real-world use of nintedanib for PF-ILD.

Many thanks for offering to submit data for this national service evaluation of the use of nintedanib in progressive fibrosing ILD.

The Service Evaluation covers the time period of 17th November 2021 to 30th September 2022.

All contributors and supervisors will be included in the authorship of publications resulting from this work. All contributors and supervisors will be invited to review any proposed publication.

Please document names and centres as you wish them to be acknowledged in any future publications.

For any queries please contact gilesdixon@nhs.net

Basic information

1. Name of specialist ILD centre ✱ *Required*

2. Name of local service evaluation lead ✱ *Required*

3. Email address of service evaluation lead ✱ *Required*

Please enter a valid email address.

4. Name of supervising consultant (if applicable)

5. Email address of supervising consultant (if applicable)

Please enter a valid email address.

Diagnosis

6. How many patients have had an ILD MDT diagnosis of PF-ILD and have been prescribed (or are awaiting prescription) of nintedanib?

More info

Please enter a whole number (integer).

6.a. Of these patients which diagnostic criteria have been fulfilled to give diagnosis

More info

	Number of patients
A relative decline in FVC% predicted of at least 10% predicted in the past 24 months.	
A relative decline in FVC% predicted of at least 5% predicted, but less than 10% predicted with worsening respiratory symptoms	
A relative decline in FVC% predicted of at least 5% predicted, but less than 10% predicted with increasing fibrotic changes on HRCT compared in the past 24 months.	
Worsening respiratory symptoms and increasing fibrotic changes on high-resolution chest imaging in the past 24 months.	
≥1 diagnostic criteria	
Other diagnostic criteria (please state below with diagnosis and number of patients)	

6.a.i. If other please state diagnosis and number of patients. e.g. MDT consensus opinion of disease progression, x patients

6.b. Of these patients what has the **primary** diagnosis been

More info

	Number of patients
Hypersensitivity pneumonitis	
RA-ILD	
SSc-ILD	

Mixed connective tissue disease associated ILD	
Anti-synthetase syndrome	
Sjogrens	
Vasculitis	
Other autoimmune ILDs	
Idiopathic NSIP	
Unclassifiable idiopathic interstitial pneumonia	
Sarcoidosis	
Exposure-related ILD	
Familial PF	
CPFE	
Other ILD	

6.b.i. If other please state diagnosis and frequency e.g. x disease, x patients

Co-prescription

7. At the time of ILD MDT diagnosis of PF-ILD and decision to offer nintedanib, how many individual patients were taking the following medications for treatment of their ILD?

More info

	Number of patients taking medication at time of initiation of nintedanib
Abatacept	
Adalimumab	
Azathioprine	
Ciclosporin	
Denosumab	
Etanercept	
Hydroxychloroquine	
Infliximab	
Leflunomide	
Oral corticosteroids	
Methotrexate	
Mycophenolate mofetil	
Rituximab	
Sulfasalazine	
Tacrolimus	
Tocilizumab	
Other relevant for treatment of PF-ILD (see below)	

7.a. If other please state drug and number of patients. e.g. x drug, y patients

8. In how many patients have the following medications (for treatment of ILD) been stopped (or paused) in order to initiate nintedanib?

	Number of patients
Abatacept	
Adalimumab	
Azathioprine	
Ciclosporin	
Denosumab	
Etanercept	
Hydroxychloroquine	
Infliximab	
Leflunomide	
Oral corticosteroids	
Methotrexate	
Mycophenolate mofetil	
Rituximab	
Sulfasalazine	
Tacrolimus	
Tocilizumab	
Other relevant for treatment of PF-ILD (See below)	

8.a. If other please state drug and number of patients. e.g. x drug, y patients

Imaging

9. How many patients had the following predominant HRCT appearances

	Number of patients
Definite UIP	
Probable UIP	
Indeterminate UIP	
Fibrotic NSIP	
Fibrotic HP	
Alternative pattern	

Pulmonary Function Testing

10. How many patients had the following % predicted FVC range at the time of MDT decision to offer nintedanib for PF-ILD

	Number of patients
<40%	
≥40 to <50%	
≥50 to <60%	
≥60 to <70%	

≥70 to <80%	
≥80 to <90%	
≥90%	

11. How many patients had the following % predicted TLCO range at the time of MDT decision to offer nintedanib for PF-ILD

	Number of patients
<40%	
≥40 to <50%	
≥50 to <60%	
≥60 to <70%	
≥70 to <80%	
≥80 to <90%	
≥90%	

Drug initiation

12. As of 30th September 2022 how many patients have commenced nintedanib for PF-ILD?

More info

12.a. Of these patients how many have discontinued nintedanib?

12.b. What were the reasons for drug discontinuation?

	Number of patients
Death	
Drug tolerability	
Deranged LFTs	
Other	

12.b.i. If other please state reason and number of patients e.g. rash, x patients

Service delivery

13. Which services are initating nintedanib for PF-ILD? (Tick all that apply)

- ☐ ILD service
- ☐ Joint rheumatology/ILD service or equivalent
- ☐ Rheumatology service independent of ILD service
- ☐ General respiratory physician
- ☐ Other

13.a. If you selected Other, please specify:

14. Who are the prescribers of nintedanib for PF-ILD in your service? (Tick all that apply)

- ☐ Pharmacist
- ☐ Respiratory physician
- ☐ Rheumatologist
- ☐ Nurse specialist
- ☐ Specialist physiotherapist
- ☐ Other (please state)
- ☐ Other

14.a. If you selected Other, please specify:

Page 2: Final page

Many thanks for completing the service evaluation data collection form.

Your contribution is highly appreciated.

Giles, Michael and Shaney
