# Supplementary material for: Real-world experience of nintedanib for progressive fibrosing interstitial lung disease in the UK
Source: ERJ Open Res. 2024 Jan 15;10(1):00529-2023. doi: 10.1183/23120541.00529-2023 (PMC10789269; doi:10.1183/23120541.00529-2023)
Supplement: Supplementary file 2 [file 00529-2023.SUPPLEMENT2.pdf]

Co-ordinating Centres

Bristol Interstitial Lung Disease Servive, North Bristol NHS Trust, Bristol, UK
Royal Devon University Healthcare NHS Foundation Trust, Exeter, UK

Participating Centres

Antrim Area Hospital, Northern Health and Social Care Trust, Antrim, Northern Ireland, UK
Glenfield Hospital, University Hospitals of Leicester NHS Trust, Leicester, UK
Guy's and St Thomas' Hospital NHS Foundation Trust, London, UK
Hammersmith Hospital, Imperial College Healthcare NHS Trust, London, UK
Hull University Teaching Hospitals NHS Trust, Hull, UK
Interstitial Lung Disease Unit, Wythenshawe Hospital, Manchester University NHS Foundation Trust, Manchester, UK
Leeds Teaching Hospitals NHS Trust, Leeds, UK
Liverpool Interstitial Lung Disease Service, Aintree Hospital, Liverpool University Hospital NHS FT, Liverpool, UK
New Cross Hospital, The Royal Wolverhampton NHS Trust, Wolverhampton, UK
Norfolk and Norwich University Hospital NHS Foundation Trust, UK
Nottingham University Hospitals NHS Trust, Nottingham, UK
Oxford University Hospitals NHS Foundation Trust, Oxford, UK
Royal Brompton and Harefield Hospitals, London, UK
Royal Infirmary of Edinburgh, Edinburgh, UK
Sheffield Teaching Hospital NHS Foundation Trust, Sheffield, UK
Southern Health and Social Care Trust, Northern Ireland, UK
St Mary's Hospital, Imperial College Healthcare NHS Trust, London, UK
The Newcastle upon Tyne Hospitals NHS Foundation Trust, Newcastle, UK
University Hospital of Southampton NHS Foundation Trust, Southampton, UK
University Hospitals Birmingham NHS Foundation Trust, Birmingham, UK
University Hospitals of Morecambe Bay NHS Foundation Trust, Lancashire and South Cumbria ILD service, Lancaster, UK
University Hospitals of North Midlands NHS Trust, Stoke-on-Trent, UK

Centres listed in alphabetical order
